# An Embedded Sensor System for Real-Time Detecting 5-DOF Error Motions of Rotary Stages

**DOI:** 10.3390/s19132855

**Published:** 2019-06-27

**Authors:** Zhi-Feng Lou, Xiu-Peng Hao, Yin-Di Cai, Tien-Fu Lu, Xiao-Dong Wang, Kuang-Chao Fan

**Affiliations:** 1 Key Laboratory for Precision and Non-traditional Machining Technology of Ministry of Education, Dalian University of Technology, Dalian 116023, China; 2 School of Mechanical Engineering, the University of Adelaide, Adelaide, SA 5005, Australia; 3 Department of Mechanical Engineering, National Taiwan University, Taipei 10617, Taiwan

**Keywords:** radial error, tilt error, rotary stage, real-time measurement

## Abstract

The geometric error motions of rotary stages greatly affect the accuracy of constructed machines such as machine tools, measuring instruments, and robots. In this paper, an embedded sensor system for real-time measurement of two radial and three angular error motions of a rotary stage is proposed, which makes use of a rotary encoder with multiple scanning heads to measure the rotational angle and two radial error motions and a miniature autocollimator to measure two tilt angular errors of the axis of rotation. The assembly errors of the grid disc of the encoder and the mirror for autocollimator are also evaluated and compensated. The developed measuring device can be fixed inside the rotary stage. In the experiments, radial error motions of two points on the axis (*h* = 5 mm and 60 mm) were measured and calibrated with LVDTs, and the data showed that the radial error motions of the axis were less than 20 μm, and the calibration residual errors were less than 2 μm. When intermittent external forces were applied to the stage, the change of the stage’s error motion could also be monitored accurately.

## 1. Introduction

Rotary stages are widely used in industrial fields, such as machine tools, robots, measuring machines, etc. Due to design, manufacturing, and assembly errors rotary stages have inherent geometric errors in six degrees of freedom (6-DOF) as described in ISO230-1, ISO230-7, and ASME B89.3.4 [1,2,3] as well as in the book by Marsh [4], which includes three translational errors and three rotational errors.

Many previous studies have proposed various measuring methods for error motions of rotary stages. Precise spheres or cylinders are usually used as reference artifacts to measure radial error motions of rotary stages. Applying error separation techniques, the influence on the measurement accuracy by the geometric errors of artifacts can be eliminated [5,6]. A general methodology of self-calibration in dimensional metrology (including the spindle errors) was addressed by Evans, who used a reversal error separation method [7].

To measure all six geometric errors one by one is a tedious work that would generate measurement uncertainty due to elapsed time. Therefore, many methods for measuring multi-degree-of-freedom (DOF) errors of rotary stages also were developed. For example, Jywe established a novel technique using diffractive grating and position sensitive detectors (PSDs) to calibrate the 4-DOF errors of a rotary table [8]. Liu used position sensitive detectors (PSDs) and diffraction grating to develop measuring devices for the error motions and the angular indexing error [9]. Sung proposed a measurement system based on a laser diode, PSDs, and the homogeneous transformation approach to measure six geometric errors of a rotary axis separately [10]. Muhummad proposed a measurement method using concentric circle grating and phase modulation interferometers to measure spindle radial, axial, and angular motions concurrently [11]. Ahn proposed a new smart sensor system that can measure the complete five-dimensional motions of a rotating shaft by combining two radial cylindrical capacitive sensor and a specially designed target rotor [12]. Murakami developed a simple and low-cost optical measurement system for the simultaneous measurement of 5-DOF error motions of high-speed micro-spindles [13]. Anandana also presented a methodology that employed two laser Doppler vibrometer systems to measure radial and tilt error motions of ultra-high-speed miniature spindles [14]. Feng’s group applied polarization technique to divide the optical path into an interferometric part and a collimation part so that 6-DOF errors are possible to be measured simultaneously [15,16]. 

In a five-axis machine tool, two axes are built by rotary stages orthogonal to each other. The accuracy of rotational motion is very important. Zhang proposed a novel measuring method for geometric error identification of the rotary table on five axis machine tools using a double ball bar [17]. Wang used a laser tracker and adopted the multi-station and time-sharing measurement principle to detect the rotary axis of NC machine [18]. A method using dual optical path of laser interferometer to one-by-one identify all 6-DOF errors of a rotary stage was proposed by He [19]. In view of above-mentioned methods, those are all instrument systems rather than sensor systems, which means they cannot be permanently embedded in the stages.

Analogous to the Abbe principle in translational motion that positioning error would be induced by angular errors if the reference axis is not in line with the functional axis, it also appears in the rotational motion that the angular indexing error would be induced by tilt and radial error motions if the functional plane, i.e., the working plane, has an offset from the encoder plane. Li proposed a novel method to analyze the angular positioning error of rotary stages based on the concepts of the Abbe principle [20]. Lou also developed an analytical model to predict the angular indexing error of a rotational mandrel with both ends supported by a two-center module in the gear measuring machine [21]. Both the above methods are, however, off-line type.

In this report, a real-time embedded measuring method for radial and tilt error motions of rotary stages is proposed, in which an encoder with multiple scanning heads is used to measure rotational angle and radial error motions, and a miniature autocollimator is used to measure tilt errors of the shaft. A novel mathematical model is proposed to eliminate the assembly errors of the encoder and mirror of autocollimator. The developed measuring device can be built into the rotary stage. It is able to detect the change of error motions when the stage bears a sudden external force.

## 2. Measurement Methodology

**Figure 1:**
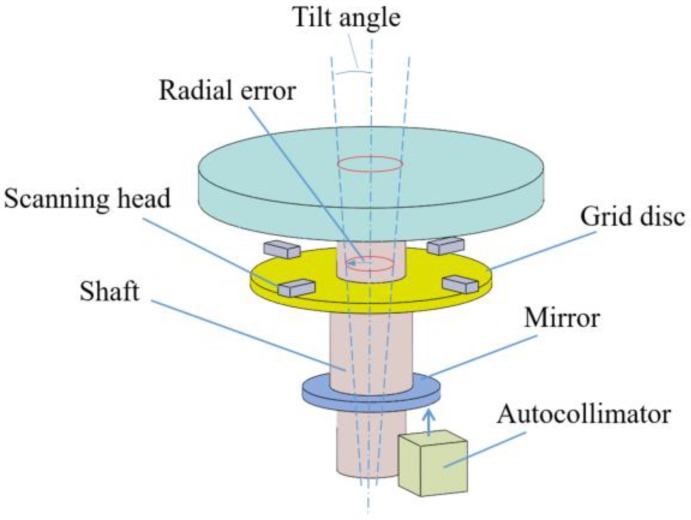
Measuring method for attitude error of the rotary stage.

In order to improve measurement accuracy of the rotational angle, an encoder with multiple scanning heads is often used in the rotary stage so that the indexing error caused by assembly eccentricity of the grid disc to the axis of rotation, or the spindle, can be removed [22]. However, the tilt error motion of the spindle is not measured and its affect to the indexing error is unknown. In analogy to the Abbe principle for linear displacement measurement, the angular indexing error must exist if the grid disc is not in the measured plane. The effect has been analyzed and verified by the author’s group [20,21]; however, the measurement method was off-line with five motion. An embedded sensor system for real-time detecting 5-DOF error motions of rotary stages is shown in Figure 1. The grid disc of the rotary encoder is equipped with four scanning read heads so that the instantaneous angle of rotation and two orthogonal eccentric motions, or called radial error motions, of the disc can be detected. In addition, a mirror disc is mounted onto the spindle, whose two tilt motions orthogonal to the axis of rotation can be simultaneously detected by a miniature autocollimator mounted on the base of the stage. Such a simple and low-cost setup allows on-line detection of 5-DOF motions of the spindle except the axial slip. Among these five error motions, the rotational indexing error has been analyzed in the author’s previous report [20,21] and is not repeated here. The measurement principles of other four error motions, namely two radial error motions and two tilt errors, will be detailed in the following sections.

### 2.1. Measurement of the Two Radial Error Motions

**Figure 2:**
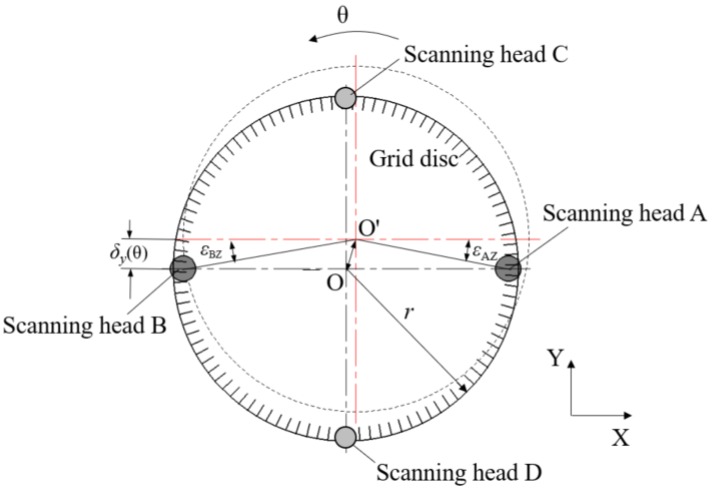
Measurement of rotation angle when error existing.

In the rotary stage, the grid disc of the encoder is fixed to the spindle shaft and rotated by the shaft. Figure 2 shows the geometrical relationship of the rotating disc and fixed four scanning heads along the X-direction and Y-direction. The solid line is the initial position of the grid disc and the dotted line is the position after rotating a *θ* angle when radial error motion of the grid disc due to assembly eccentricity exists. It is apparent for scanning heads A and B that an in-plane shift of the disc’s measured point in Y-direction occurs, $\delta_{y}\left(\theta \right)$, with respect to its corresponding scanning head.

As shown in Figure 2, after rotating a *θ* angle from initial position, the grid disc has radial displacement *δ*_Y_(*θ*) in the Y-direction. According to geometric analysis, the indications of the scanning heads A and B (*θ*_A_ and *θ*_B_) can be expressed as follows.

(1) $$\theta_{A}=\theta +\varepsilon_{\mathrm{AZ}}\left(\theta \right)=\theta +\tan^{-1}\left[\delta_{y}\left(\theta \right)/r\right],$$

(2) $$\theta_{B}=\theta -\varepsilon_{\mathrm{BZ}}\left(\theta \right)=\theta -\tan^{-1}\left[\delta_{y}\left(\theta \right)/r\right],$$

where, $\varepsilon_{\mathrm{AZ}}\left(\theta \right)$ and $\varepsilon_{\mathrm{BZ}}\left(\theta \right)$ are induced indexing error at sensing points A and B, respectively, and r is the radius of the grid disc.

According to Equations (1) and (2), the displacement of the grid disc’s center in the Y-direction can be expressed by

(3) $$\delta_{y}\left(\theta \right)=\tan\left(\frac{\theta_{A}-\theta_{B}}{2}\right)\times r,$$

Obviously, this radial error motion in the Y-direction includes the error caused by assembly eccentricity of the grid disc $\delta_{y1}\left(\theta \right)$ and actual radial error motion of the axis $\delta_{y2}\left(\theta \right)$,

(4) $$\delta_{y}\left(\theta \right)=\delta_{y1}\left(\theta \right)+\delta_{y2}\left(\theta \right),$$

Similarly, the other two scanning heads oppositely fixed in the Y-direction yield

(5) $$\delta_{x}\left(\theta \right)=\delta_{x1}\left(\theta \right)+\delta_{x2}\left(\theta \right),$$

where, $\delta_{x}\left(\theta \right)$ is the radial error motion of the grid disc in the X-direction, $\delta_{x1}\left(\theta \right)$ is the error caused by assembly eccentricity of the grid disc, and $\delta_{x2}\left(\theta \right)$ is the actual radial error motion of the axis. Hence, $\delta_{x2}\left(\theta \right)$ and $\delta_{y2}\left(\theta \right)$ can be obtained if errors caused by assembly eccentricity of the grid disc are known.

**Figure 3:**
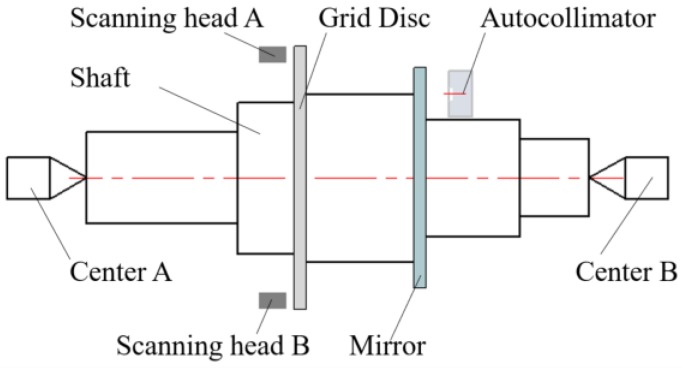
Measurement of assembly error.

During the assembly of the whole rotary stage, the grid disc has to be assembled to the shaft before assembling the shaft into the rotary stage. The eccentricity error of the grid disc to the shaft can be measured in the initial stage, as shown in Figure 3. The shaft with the grid disc is supported by two centers and driven to rotate. Two scanning heads are fixed symmetrically to measure corresponding rotational angles. Because no radial error motion of the shaft exists at this stage, Equation (3) simply results in the assembly eccentricity error of the grid disc $\delta_{y1}\left(\theta \right)$. Hence, after the whole stage is assembled and tested again, $\delta_{y}\left(\theta \right)$ can be calculated by Equation (3) again so that $\delta_{y2}\left(\theta \right)$ can be separated by Equation (4). The same process can be applied to the x component in Equation (5). As a result, all $\delta_{y1}\left(\theta \right)$, $\delta_{y2}\left(\theta \right)$, $\delta_{x1}\left(\theta \right)$ and $\delta_{x2}\left(\theta \right)$ are measurable from this proposed method.

### 2.2. Measurement of the Two Tilt Error Motions

As shown in Figure 1, a miniature autocollimator was used to measure the tilt error motion of the rotary stage, but such a measured tilt error (ε) contains the slope of the flatness of the mirror (α) and the actual tilt error motion of the axis ($\varepsilon_{s}$), which has to be removed.

(6) $$\varepsilon \left(\theta \right)=\alpha \left(\theta \right)+\varepsilon_{s}\left(\theta \right),$$

Based on the similar principle to Figure 3, the shaft with the mirror is supported by the two centers and the autocollimator, which is fixed to the base plate, directly measures the assembly tilt and flatness error of the mirror when the axis rotates to different angular positions. In other words, the error of α(θ) can be measured in advance during the sensor assembly process. After the whole stage is assembled, the scheme in Figure 1 provides the value of ε(θ). Substituting ε(θ) and α(θ) into Equation (6), the actual tilt error motion of the shaft $\varepsilon_{s}\left(\theta \right)$ can be obtained.

**Figure 4:**
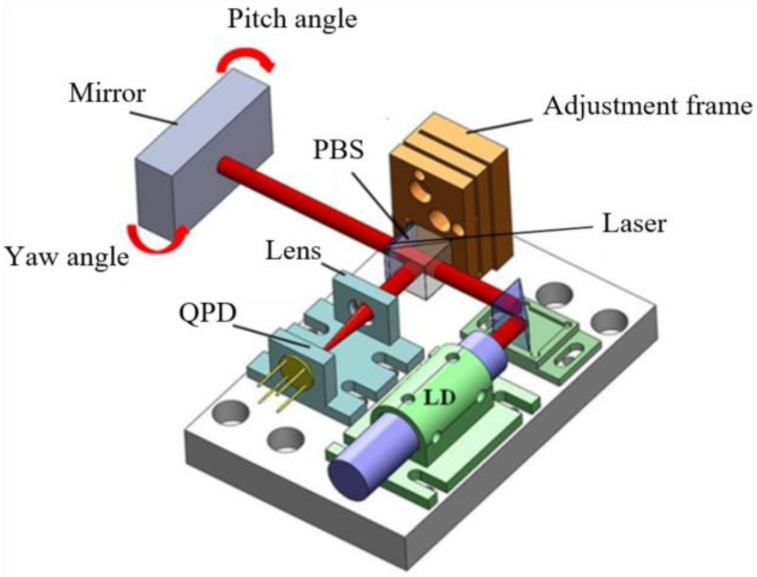
Laser auto-collimator schematic.

The principle of the miniature autocollimator and its design and fabrication can be found in the author’s previous report [23]. A concise optical system is shown in Figure 4. A laser beam passes through a polarization beam splitter (PBS) and a 1/4 wave plate, and enters to a mirror. The reflected beam passes through the 1/4 wave plate and the PBS again and is focused on a quadrants photo detector (QPD) after passing a focusing lens. If the mirror has tilt motion, the position of the focused spot on the QPD will change, so that the title angle is measured.

**Figure 5:**
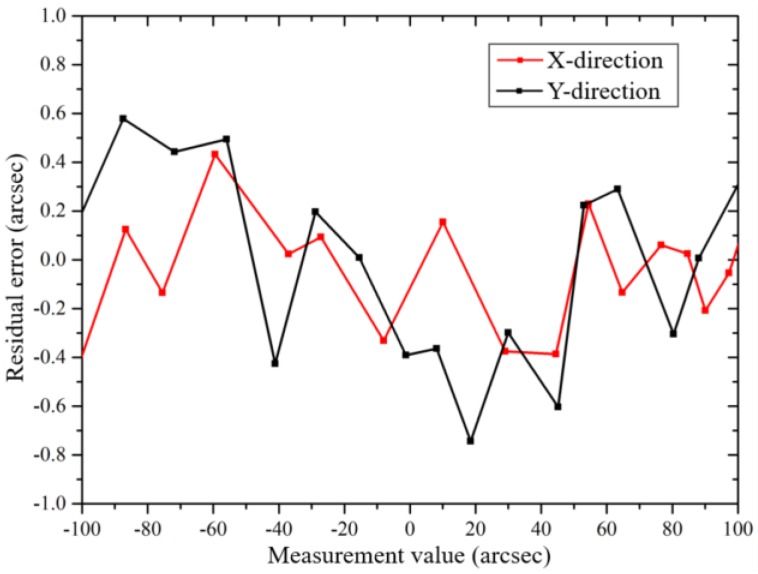
Calibration of the laser autocollimator.

Figure 5 shows the calibrated results of the miniature autocollimator by a commercial photoelectric autocollimator (AUTOMAT1000UH-3050, the accuracy is ±0.2 arcsec). Residual errors are ±0.5 arcsec and ±0.8 arcsec in the directions of X and Y, respectively, within the measurement range of ±100 arc-second with a resolution of ±0.1 arcsec.

## 3. Experiments

**Figure 6:**
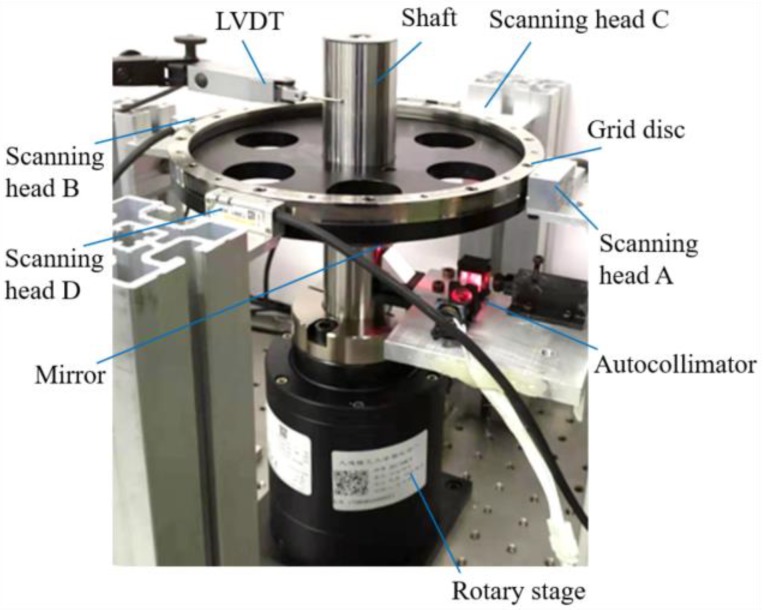
Experimental setup.

The experimental setup is shown in Figure 6. An encoder (Renishaw RESM20-USA200, the disc’s radius is 200 mm, and the resolution is 0.412 arcsec) with four scanning heads was used to measure rotational angle and radial error motions of the shaft. A LVDT was used to verify the predicted radial motion at a certain height. According to Equation (3) and the resolution of the encoder, the resolution of the measured radial error is 0.10 μm. The small autocollimator was fixed under the grid disc to measure the tilt error motion of the axis of rotation. Measurement data of the autocollimator and the encoder are acquired in real time by a multifunction I/O device (NI USB6002, sample rate is 50 kS/s) and two motion control devices (Lechuang MPC08) respectively. According to the sample rate, sample interval is 25.9 arcsec when the rotation speed of the stage is 1r per second in the experiment. If smaller latency is required, the data acquisition device with higher sample rate should be used. 

### 3.1. Measurement of Original Errors

**Figure 7:**
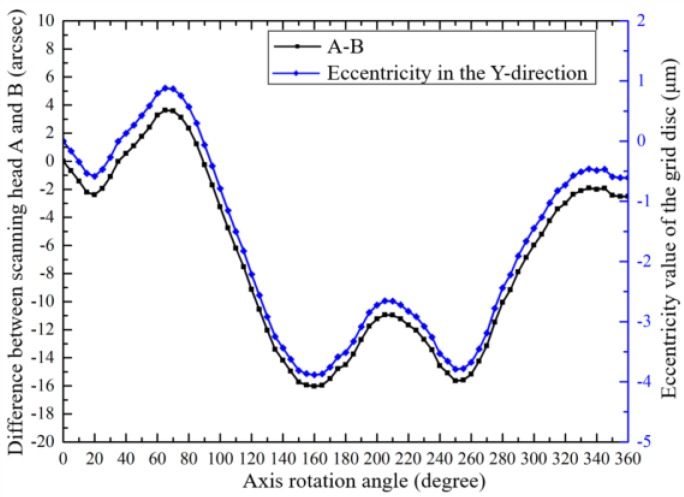
The difference between the two scanning heads’ indications and assembly eccentricity of the grid disc.

The assembly error of the grid disc was measured according to Figure 3. Figure 7 shows the indication difference (*θ*_A_ − *θ*_B_) between the two scanning heads when the shaft rotated in different positions of a complete cycle. The assembly eccentricity error of the grid disc in the Y-direction (*δ*_y_(*θ*)) can be computed according to Equation (3), which also is shown in Figure 7. 

**Figure 8:**
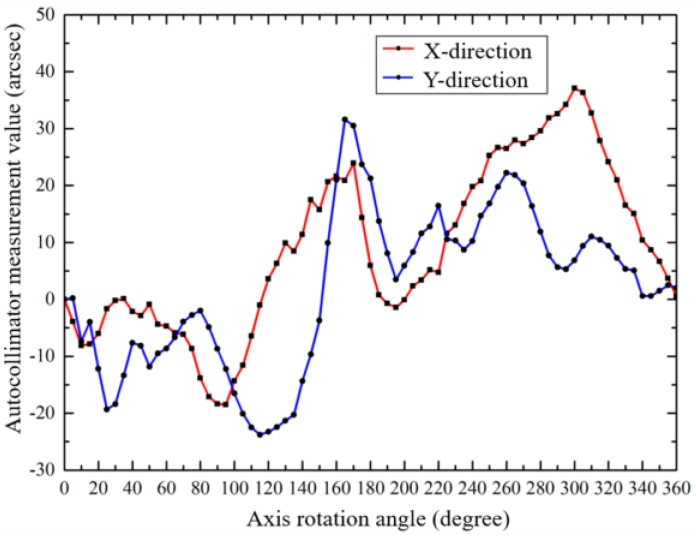
The tilt error of the mirror.

The tilt errors caused by assembly error and flatness error of the mirror were measured and shown in Figure 8. 

### 3.2. Measurement of the Axis Rotational Indexing Error

**Figure 9:**
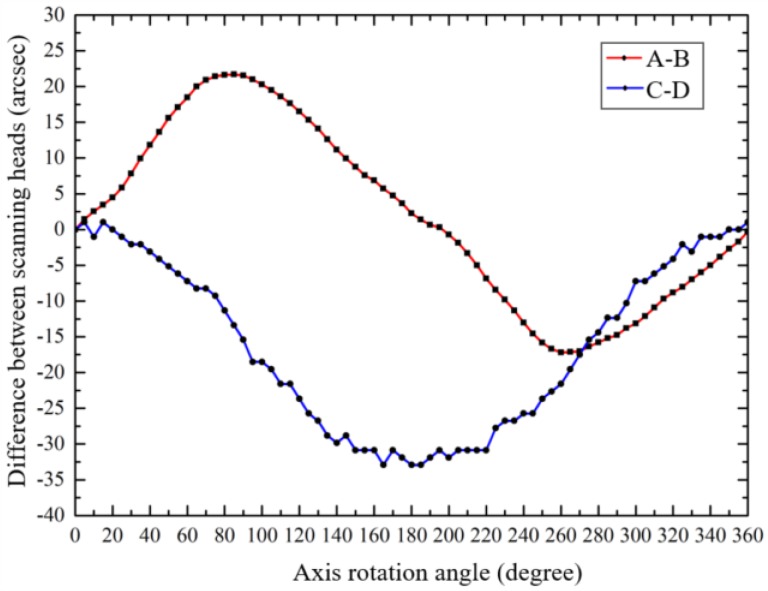
The differences between the indications of the different scanning heads.

As shown in Figure 6, the axis is driven to rotate, and four scanning heads measured the rotational indexing angle of the axis. The indication differences between different scanning heads, in X-direction (A-B) and Y-direction (C-D), are shown in Figure 9.

**Figure 10:**
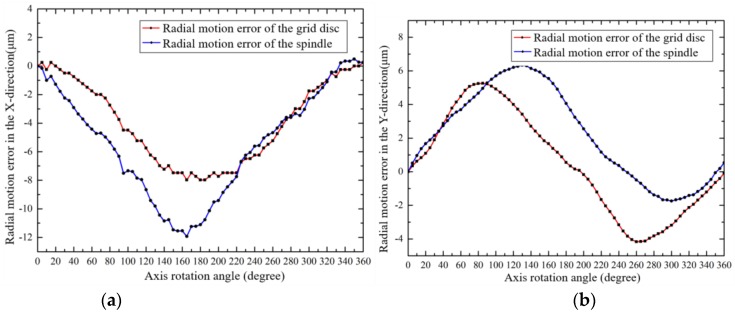
The radial error motions of the grid disc and the shaft: (**a**) X-direction; (**b**) Y-direction

According to Equations (3) to (5) and the data in Figure 7, errors caused by the assembly eccentricity of the grid disc were removed, and both the radial error motions of the grid disc and the shaft are shown in Figure 10. 

**Figure 11:**
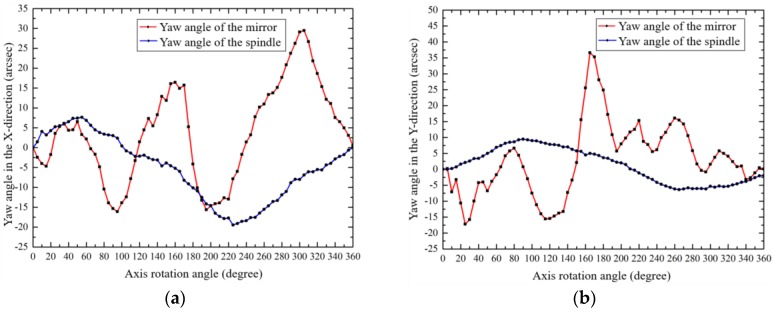
The motion tilt error of the mirror and the shaft: (**a**) X-direction; (**b**) Y-direction.

The total tilt errors in X and Y directions were measured by the autocollimator according to Figure 1, and mirror tilt errors were measured according to Figure 3. Figure 11 shows the measured mirror tilt errors and the separated actual shaft tilt errors calculated by Equation (6).

### 3.3. Verification 

**Figure 12:**
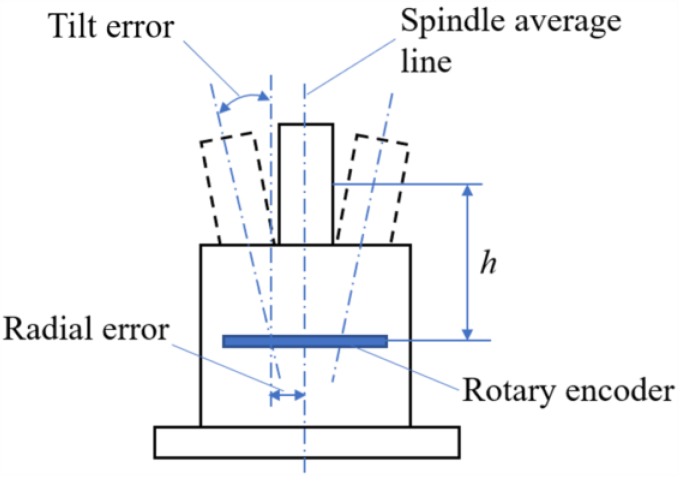
The radial error motions of different points on the axis.

The radial error motion of one particular point on the shaft can be computed according to Equation (7) if the tilt error of the shaft has been obtained. The schematic diagram of testing the radial error motion of the point offset from the grid disc a distance h is shown in Figure 12. The measured error can be compared with the following predicted error based on the following equation.

(7) $$\delta_{h}=\delta +h\times \tan\varepsilon_{s},$$

In Equation (7), *δ_h_* is the radial error of the point on the axis of which offset distance from the grid disc is *h*, *δ* is the shaft radial error at the position of the grid disc, and $\varepsilon_{s}$ is the actual tilt error angle of the shaft.

**Figure 13:**
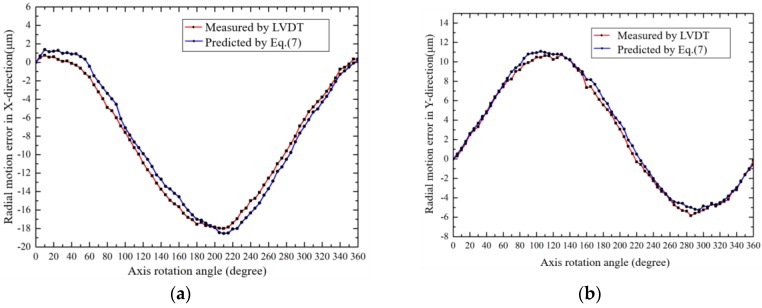
The radial error motion of the axis (*h* = 5 mm): (**a**) X-direction; (**b**) Y-direction.

**Figure 14:**
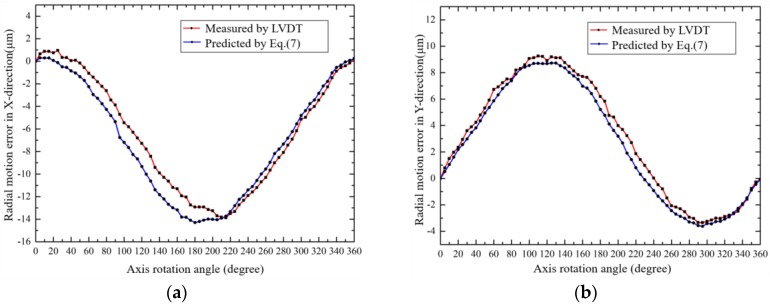
The radial error motion of the axis (*h* = 60 mm): (**a**) X-direction; (**b**) Y-direction.

In order to verify above analysis and measurement, radial error motions of two points on the axis (*h* = 5 mm and 60 mm in Figure 12) were measured by a LVDT (Mahr Millimar-1240 with probe 1318, the accuracy is ±0.1 μm). The comparison data are shown in Figure 13 and Figure 14, respectively. It was found that the radial error motions of the axis were less than 20 μm and the residual error was less than 2 μm, which verifies that the developed analysis and measurement method is accurate and feasible. 

The rotatory stages are usually used in machine tools, or robots, or measuring instruments. The stage or the bearings will be deformed when external force is applied, and error motion will be different from static or non-force state. By using the measuring method proposed in this study, the error motion of the axis or stages can be monitored in real-time, and errors of manufacture or measurement can be compensated. 

**Figure 15:**
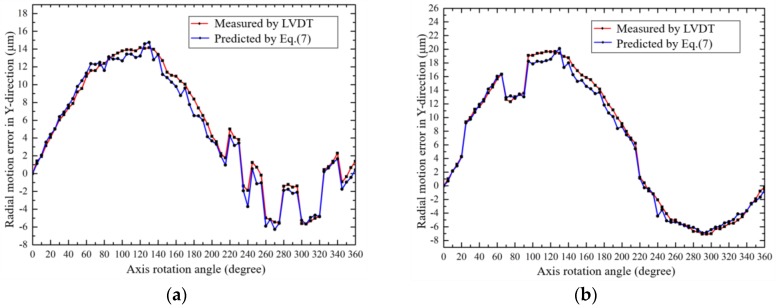
Verification experiments when the external forces were applied on the shaft: (**a**) the 1st time; (**b**) the 2nd time.

In the experiments, a heavy mechanical part was fixed onto the top of the shaft. During the rotation of the shaft, intermittent external forces were applied to the mechanical part. Such a disturbance would cause reactive error motions of the shaft, including radial error motions and tilt errors; both were measured by the developed embedded sensors. The radial error motions of the axis in the Y-direction at an offset distance (*h* = 16 mm) from the grid disc were predicted by the proposed model and verified by the LVDT. The measured data are shown in Figure 15, and it can be seen that not only the error trend but also its magnitude at any position are very consistent. The correctness of the proposed method is verified.

## 4. Conclusions

A novel method for real-time simultaneously measuring two radial error motions, two tilt error motions, and one angular indexing error of the rotary stage has been developed in this paper, being an embedded sensor system for five-DOF errors. It used an encoder with multiple scanning heads to measure rotational angle and radial error motions of the shaft and a miniature autocollimator to measure tilt angular errors of the shaft. From the detected radial and tilt errors, the angular indexing error can be evaluated, which has been verified in the authors’ previous report. In this report, a mathematical model to analyze the actual tilt errors of the shaft and radial error motion of any point offset from the grid disc has been proposed. The assembly errors of the grid disc and the mirror were measured and removed. The radial error motions at the different points on the shaft were measured and verified by the LVDT, and the residual error was less than 2 μm. The developed method can easily be used to monitor the error motion of the rotary stage in real-time, even if error motions change when the stage bears external force. The developed embedded sensor system provides error compensation signals of the machine constructed by the developed rotary stage. This will be our future work.
